# Supplementary material for: Complexome profiling of the Chlamydomonas psb28 mutant reveals TEF5 as an early PSII assembly factor
Source: Plant Cell. 2025 Mar 17;37(6):koaf055. doi: 10.1093/plcell/koaf055 (PMC12164590; doi:10.1093/plcell/koaf055)
Supplement: koaf055_Supplementary_Data [file koaf055_supplementary_data.zip › psb28_interactive.zip!/plots/intensity/qualityPlots/peptides/_Cre03.g164300.t1.2.html]

### Cre03.g164300.t1.2

Trivial name:   
SeqCoverage [median]: 29.00  
Mass: 13.33 kDa  
  
 

| Sequence | MissedCleavage | Mass [Da] | wtIntensity | muIntensity | Score |
| --- | --- | --- | --- | --- | --- |
| DNSGRQNIFPTSSK | 1 | 1549.75 | 13572.35 | 21825.85 | 178.67 |
| QGDETLAQVNNAYQGDSLTVISSR | 0 | 2565.23 | 990.30 | 0.00 | 85.57 |
| QNIFPTSSK | 0 | 1020.52 | 29974.64 | 29181.54 | 158.61 |
|  |  |  | 44537.3 | 51007.4 |  |
